# Asymmetric stimulus representations bias visual perceptual learning

**DOI:** 10.1167/jov.24.1.10

**Published:** 2024-01-29

**Authors:** Pooya Laamerad, Asmara Awada, Christopher C. Pack, Shahab Bakhtiari

**Affiliations:** 1 Department of Neurology and Neurosurgery, Montreal Neurological Institute, McGill University, Montreal, Canada; 2 Department of Psychology, Université de Montréal, Montreal, Canada; 3 Mila – Quebec AI Institute, Montreal, Canada

**Keywords:** perceptual learning, bias, motion perception, optic flow

## Abstract

The primate visual cortex contains various regions that exhibit specialization for different stimulus properties, such as motion, shape, and color. Within each region, there is often further specialization, such that particular stimulus features, such as horizontal and vertical orientations, are over-represented. These asymmetries are associated with well-known perceptual biases, but little is known about how they influence visual learning. Most theories would predict that learning is optimal, in the sense that it is unaffected by these asymmetries. However, other approaches to learning would result in specific patterns of perceptual biases. To distinguish between these possibilities, we trained human observers to discriminate between expanding and contracting motion patterns, which have a highly asymmetrical representation in the visual cortex. Observers exhibited biased percepts of these stimuli, and these biases were affected by training in ways that were often suboptimal. We simulated different neural network models and found that a learning rule that involved only adjustments to decision criteria, rather than connection weights, could account for our data. These results suggest that cortical asymmetries influence visual perception and that human observers often rely on suboptimal strategies for learning.

## Introduction

**Figure 1.**
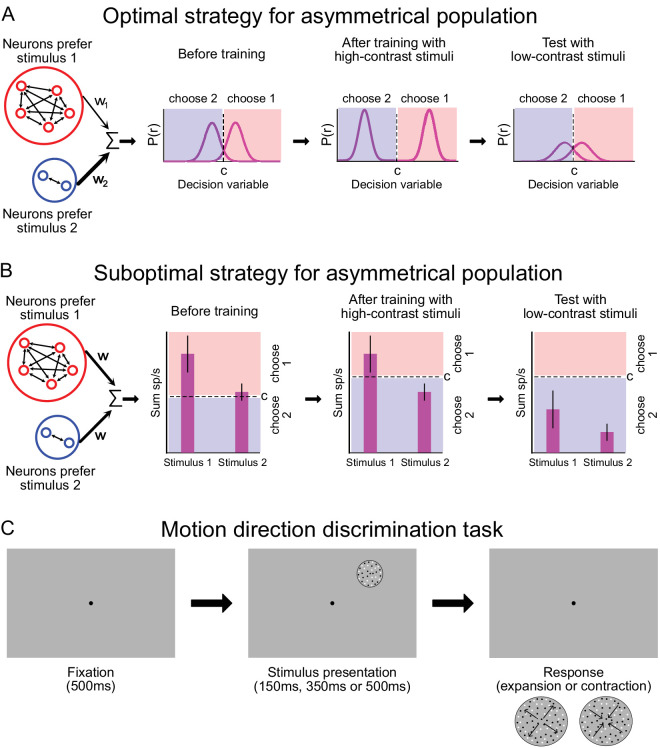
Schematics for the alternative readout strategies and the experimental procedure. (**A**) The schematics of perceptual decision-making using an optimal strategy. In an asymmetrical population (left), more neurons are selective for one stimulus condition (red) than for the other (blue). The population consists of neurons with a variety of sensitives and tuning properties to the two alternatives. Readout of the neuronal responses involves selecting relevant sensory signals that contribute to making a decision by weighting neurons based on their selectivity and summing the weighted outputs to compute the decision variable. This strategy can compensate for the asymmetry during readout, by assigning higher weights to stimulus with weaker representation or by selecting equal numbers of neurons from each pool. Training can alter the responses or the readout weights (center), thus preventing developing biased perceptual decisions (right). (**B**) An alternate strategy is to sum the total population response, with equal readout weights for all neurons, yielding a scalar measure of the likelihood of one stimulus being present (left). During training, observers can adjust their decision criterion according to the properties of the stimuli (center), but this approach will yield consistent perceptual biases when the stimulus strength changes (right). (**C**) Motion direction discrimination task used in the pretraining (one session), training (nine sessions) and post-training (one session) phases of the experiment. Each trial began when a fixation target appeared for 500 ms. Depending on the task's difficulty, the stimulus appeared for 150 ms, 300 ms, or 500 ms. The stimulus was contracting or expanding optic flow motion. Two options appeared on the screen when the stimulus and fixation target disappeared. The participant could report the direction of the motion (expansion vs. contraction) with the keyboard.

The ability to discriminate between different visual stimuli is thought to depend on their visual cortical representations: Discrimination is easiest for stimuli that yield very different response patterns in neuronal populations (Figure 1A) (Ahissar & Hochstein, 2004; Panzeri, Harvey, Piasini, Latham, & Fellin, 2017). Training-induced improvements in perceptual abilities, known as visual perceptual learning (VPL), have been suggested to arise from adjustments in sensory neuron tuning (the retuning hypothesis) (Karni & Sagi, 1991; Wenliang & Seitz, 2018)) or adjustment in the readout weights of the sensory neurons (the reweighting hypothesis) (Lu & Dosher, 2022; Sotiropoulos, Seitz, & Series, 2011)). Both theories assume optimality in VPL, which is to say that they involve learning that maximizes discrimination performance for a trained task.

In certain perceptual situations, suboptimal learning strategies could also lead to perceptual improvements. Specifically, if the population response is asymmetrical, so that one stimulus yields higher neuronal responses than another (Figure 1B), learning can proceed by adjustment of the decision criterion, without the need for retuning or reweighting of sensory responses. Such asymmetrical population responses are very common in visual cortex. Examples include stronger responses for cardinal orientations (Levick & Thibos, 1982; Nikara Bishop, & Pettigrew, 1968; Schall, Vitek, & Leventhal, 1986; Vidyasagar & Urbas, 1982), for centrifugal motion stimuli (Albright, 1989), for horizontal disparities (DeAngelis & Uka, 2003), and for circular shapes (Dumoulin & Hess, 2007). A particularly clear example of this kind of asymmetry is the over-representation of expansion optic flow compared to other optic flow patterns in the medial superior temporal (MST) area of visual cortex (Duffy & Wurtz, 1995; Graziano, Andersen, & Snowden, 1994; Mineault, Khawaja, Butts, & Pack, 2012; Saito et al., 1986).

Observers can change decision criteria easily when it is warranted by the task (Aberg & Herzog, 2012). However, this strategy is suboptimal under most conditions, because it is vulnerable to producing biased perceptual decisions when the stimulus strength deviates from that of the trained condition (Figure 1B). For example, training with a high-contrast stimulus might cause observers to increase their decision criteria, leading to perceptual biases for low-contrast stimuli. We, therefore, wondered whether observers would rely on suboptimal strategies when training with stimuli that have asymmetric cortical representations.

To answer this question, we trained a group of human observers on an optic flow two-alternative forced choice task that involved distinguishing between expansion and contraction optic flow. We found that observers exhibited biases that were predictable from the characteristics of the asymmetrical representation in MST and that these biases evolved with training in a way that was consistent with a suboptimal learning strategy.

To understand these dynamics, we compared neural networks that were trained to perform the same psychophysical task with different learning rules. Among them, only a suboptimal learning rule involving the adjustment of decision criteria was able to account for the learning sensitivity and biases observed in humans both before and after training. These results suggest that asymmetric representations of the kind that are commonly found in the primate cortex can predict the properties of visual learning.

## Methods

### Observers and apparatus

Fifty-six observers with normal or corrected-to-normal vision participated in this study (20 male observers, 36 female observers; age, 23.2 ± 3.36 years; range, 18–32 years). All observers were naïve to the purpose of the study and to visual psychophysics. Observers gave written, informed consent before their participation, and the study was approved by the Ethics Committee of the Montreal Neurological Institute and Hospital (NEU-06-033).

The experiment was run remotely and was controlled by a browser-based program (Article 19 Group; Montreal) that displayed the stimuli, monitored performance, and stored the data. Participants completed the study at home, on personal computers. Before the start of the experiment, participants were asked to provide the experimenter with their screen size. All visual displays were in the range of 13 to 27 inches diagonally and had a refresh rate of at least 30 Hz. Stimulus parameters were calibrated to each observer's screen size. The viewing distance for each participant was adjusted according to their individual screen sizes. The observers were asked to measure their distance to the screen every day and sit at the instructed distance before beginning the training session.

### Experimental procedure

The stimulus used in each experiment was an optic flow stimulus composed of an expanding or contracting random dot kinematogram. The stimulus was presented on a gray background in the upper right quadrant at an eccentricity of 6°. The random dot kinematogram was composed of small (0.06°) black dots in a 3° radius aperture with a dot density of 2.6 dots/deg^2^. Dot velocity was set to 20°/sec. Stimulus duration and dot time (after which the trajectory of the dot ended and was restarted at a random position) varied depending on the task's difficulty. The dots presented were either “signal dots” or “noise dots.” Signal dots moved coherently in a specific direction, whereas noise dots moved in random directions. The coherence of the random dot kinematogram stimulus refers to the proportion of signal dots.

Each trial started with a fixation point that the observer had to fixate for 500 ms. After the stimulus presentation (duration variable depending on the experimental condition), the fixation point and stimulus disappeared, and two options appeared on the screen (expansion and contraction). The observer was required to input their response with the keyboard (left for expansion, right for contraction). A green flash signaled a correct response and a red flash signaled an incorrect response. The direction of motion of the stimulus was chosen randomly for each trial.

As noted in the Introduction, we were interested in studying learning for different levels of task difficulty. Given the remote nature of the experiments, we were unable to precisely control stimulus contrast, which in any case does not matter much for optic flow tasks (Morrone, Burr, & Vaina, 1995). We, therefore, used stimulus duration to modulate task difficulty. For condition 1 (20 observers), the stimulus was shown for 150 ms with a dot lifetime of 75 ms. For condition 2 (19 observers), the stimulus was shown for 350 ms with a dot lifetime of 175 ms. For condition 3 (17 observers), the stimulus was shown for 500 ms with a dot lifetime of 250 ms. Each condition consisted of three phases: a pretraining phase, a training phase, and a post-training phase. In every phase of the experiment, the observers completed a direction discrimination task in which they reported the direction of the motion of the stimulus (contraction or expansion) with the keyboard (Figure 1C). Dot coherence was adjusted differently in each phase, as described elsewhere in this article.

### Experimental phases

#### Pretraining

The pretraining session required observers to report the direction of the motion of the stimulus at different coherence levels. The coherence levels tested were 0.025, 0.05, 0.1, 0.15, 0.2, 0.5, and 0.9. The pretraining session was composed of 490 trials. Each block of 70 trials tested the same coherence level. The order of coherences tested varied randomly for each participant. The pretraining session lasted approximately 30 minutes.

#### Training

The training phase ran over 9 days and required one training session to be completed per day. One training session was composed of four blocks of training. Each block was composed of 125 trials. The observers were compensated with 1.2 cents (Canadian) per correct response. At the start of each block, the initial coherence of the stimulus was set to 0.7. The coherence for each subsequent trial was set using a two-down–one-up adaptive staircase procedure, resulting in an 83% convergence level (Leek, 2001). Observers were allowed to take a break between each block. Each daily training session lasted approximately 30 minutes.

#### Post-training

The procedures for the post-training were the same as for the pretraining. The only difference was that observers received no feedback for correct and incorrect responses, in order to avoid further training effects. The order of coherences tested was chosen randomly and differed from the order of coherences tested in the pretraining phase.

### Data analysis

#### Psychometric curve fitting

The observers’ performance as a function of coherence was characterized by fitting a Weibull function to the proportion of correct responses using the maximal likelihood algorithm (MATLAB Palamedes toolbox for analyzing psychophysical data) (Prins & Kingdom, 2018). The Weibull function is given as:

(1) $$\begin{array}{c} F_{W}\left(x;\;\alpha ,\;\beta \right)=1-ex\left(-{\left(\frac{x}{\alpha }\right)}^{\beta }\right),\; \end{array}$$

where α determines the threshold and parameter β corresponds with the slope of the function. The criterion of maximum likelihood was used to find the best fitting psychometric function to each observer's performance.

#### Bias calculation

We used the equation below to calculate the bias (Wickens, 2001):

(2) $$\begin{array}{ccc} \;(Bias)\; & = & \left(Norminv^{2}\left(Expansion\;hit\;rate\right)\right.\\ \; & & -\left.\;Norminv^{2}\left(Contraction\;hit\;rate\right)\right)/2\; \end{array}$$

where *Norminv* is the normal inverse cumulative distribution function. A log-transform was used to remove a nonlinear effect of bias. Negative bias values indicate a bias toward contraction, and positive values indicate a bias toward expansion.

#### Statistical comparisons

Statistical comparisons of computed bias values were based on the one-tailed Wilcoxon signed-rank (WSR) test. To calculate the statistical difference of biases before and after the training, we used the Wilcoxon rank sum (WRS) test.

#### Model

To determine how observer learning might have progressed, we simulated four computational models, each starting from the assumption that psychophysical decisions were based on the asymmetrical representation found in area MST. The three models embodied different optimal and suboptimal learning rules. The equation below describes the model:

(3) $$\begin{array}{c} \;y=\sigma \left(g^{e}\sum_{i=1}^{M_{e}}w_{i}^{e}x_{i}^{e}+g^{c}\sum_{i=1}^{M_{c}}w_{i}^{c}x_{i}^{c}+c_{d}\right),\; \end{array}$$

where $x_{i}^{e}$ and $x_{i}^{c}$ are the activation of the expansion and contraction neurons in MST, respectively. $w_{i}^{e}\;w_{i}^{c}$ are their readout weights (the strength of synaptic connections to the readout neuron), *g^e^* and *g^c^* are sensory gains, *c_d_* is the decision criterion (or the bias term), and *y* represents the activation of the readout neuron. In different versions of our models, the tuning properties of the neurons are fixed, and the readout weights ($w_{i}^{e}$,$w_{i}^{c}$), the decision criterion (*c_d_*), and the sensory gains (*g^e^*,*g^c^*) are the only model parameters that change. All the trainable parameters of the model (θ) were optimized by using the gradient descent learning rule:

(4) $$\begin{array}{c} \theta^{i}=\theta^{i}-\alpha \frac{\partial L\left(\theta \right)}{\partial \theta },\; \end{array}$$

where *L* is the error rate of the model which we quantified using a binary cross-entropy loss:

(5) $$\begin{array}{c} L=\sum_{i=1}^{N}y_{i}log\hat{y_{i}}+\left(1-y_{i}\right)log\left(1-\hat{y_{i}}\right),\; \end{array}$$

where yi^s and *y_i_*s are the model outputs and the ground truth labels, respectively for the *i^th^* trial.

All the synaptic weights ($w_{i}^{e}$, $w_{i}^{c}$) were initialized to one, assuming that, at initialization, there is no a priori assumption about the relative importance of the sensory neurons in the task. The decision criterion *c_d_* was initialized to zero, and the sensory gains (*g^e^*, *g^c^*) were initialized to one.

The output of each sensory neuron ($x_{i}^{e}$, $x_{i}^{c}$) was modeled as a sigmoid function:

(6) $$\begin{array}{c} x=\frac{1}{1+e^{-k\left(s-s_{0}\right)}}.\; \end{array}$$

For the expansion and contraction neurons, the parameter *k* was set to model their opposite input selectivities (*k* = ± 5, *s*_0_ = −0.5). Variability across sensory neurons was modeled by adding random gaussian noise to *k* and *s*_0_ (ɛ∼N(0,0.2)).

## Results

### Human experiment

We trained 56 human observers to distinguish between contracting and expanding optic flow motion over the course of 9 sessions and measured their perceptual bias before and after the training. To distinguish between different learning and readout strategies (optimal vs. suboptimal), we divided the observers into three groups, each with a different stimulus duration (150, 350, and 500 ms). Stimulus duration modulated task difficulty, as shown below. Observers from all three conditions were tested before training (pre-training test) and after training (post-training test) to examine how perceptual learning changed their perceptual sensitivity and bias.

**Figure 2.**
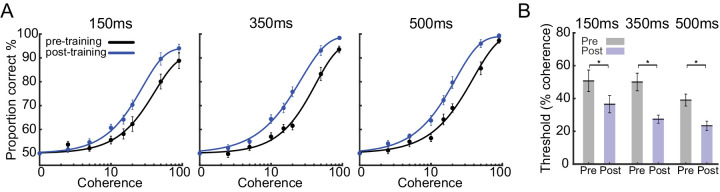
Training improved the performance of observers. (**A**) Average psychometric functions of observers in each condition pre and post training in all three conditions. The observers’ performance as a function of coherence was characterized by fitting a Weibull function to the proportion of correct responses using the maximal likelihood algorithm. (**B**) The mean psychophysical coherence threshold before and after the training in three different conditions. Under all three conditions, observers’ thresholds were significantly reduced after the training. Threshold was defined as 82% correct of the Weibull function fits. *Statistically significant difference. Error bars show standard error of the mean (SEM).

In all three duration conditions, training improved discrimination accuracy, as measured from a comparison between the mean performance on the pretraining and post-training sessions (Figure 2A). Across observers, the threshold for accurate performance (82%) was significantly reduced after the training in all three conditions (*p* = 0.02 for a 150-ms task, *p* < 0.001 for a 350-ms task, and *p* = 0.004 for the 500-ms task, *t*-test) (Figure 2B). We selected the 82% correct threshold based on the criteria defined in a previous study by Chowdhury and DeAngelis (2008).

To gain a more detailed understanding of how perceptual behavior changed in our observers, we analyzed two key aspects of their psychometric behavior: perceptual bias and the slope of the psychometric curve. To determine each observer's perceptual biases, we first determined the proportion correct for both expansion and contraction stimuli. Having no bias would mean an equal tendency to choose expansion and contraction, with any deviation from this balance indicating a non-zero perceptual bias (see Methods for detail).

**Figure 3.**
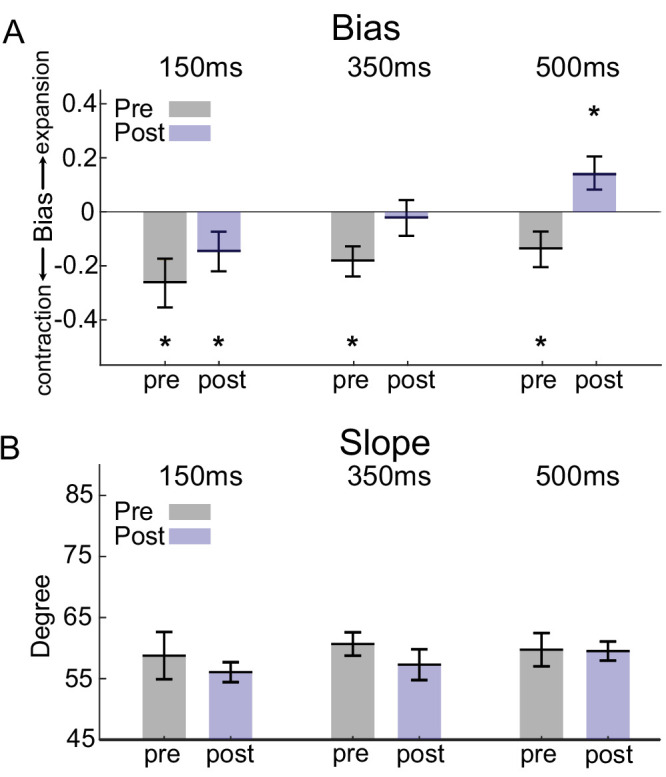
Changes in behavioral bias and slope of psychometric behavior before and after training. (**A**) The average bias across observers in each condition, before and after training. Before training, the average bias in all conditions was significantly toward contraction. This contraction bias changed after training, with the average bias in the 500 ms condition significantly shifted toward an expansion bias. Negative bias values indicate a bias toward contraction, and positive values indicate a bias toward expansion. (**B**) The mean slope of psychometric behavior among observers did not change significantly pre and post-training across all conditions. *Statistically significant difference. Error bars show standard error of the mean (SEM).

Figure 3A illustrates the biases of the observers pretraining and post-training for each stimulus strength condition. In all conditions, observers showed a significant bias toward contraction before the training (150 ms (pretraining): mean of biases ± SEM = −0.263 ± 0.88; *p* = 0.0207; 350 ms (pretraining): mean of biases ± SEM = −0.183 ± 0.055, *p* = 0.022; 500 ms (pretraining): mean of biases ± SEM = −0.138 ± 0.066, *p* = 0.0245, one-tailed WSR test) (Figure 3A). After training, these biases were unchanged in the 150-ms condition (mean of biases ± SEM = −0.147 ± 0.072; *p* = 0.0207; one-tailed WSR test), but were eliminated in the 350 ms condition (mean of biases ± SEM = −0.022 ± 0.065, *p* > 0.05; one-tailed WSR test). Interestingly, for the 500-ms condition, a significant bias toward expansion emerged with training (mean of biases ± SEM = 0.143 ± 0.061, *p* = 0.0245, one-tailed WSR test). Overall, in the 350-ms and 500-ms conditions, training resulted in a significant shift in bias (350 ms, a difference of biases = 0.161, *p* = 0.048; 500 ms, a difference of biases = 0.281, *p* = 0.0318, WRS test), whereas in the 150-ms condition there was no significant change (150 ms, difference of biases = 0.116, *p* > 0.05, WRS test). Thus, training altered observers’ biases in different ways, depending on task difficulty.

We also compared psychometric curve slopes before and after training. Any variation in the slope signifies a change in observer sensitivity to motion perception. However, on average, we observed no significant shift in slope for any of the three conditions (Figure 3B) (*p* > 0.05 for all three conditions, WRS test).

The observed patterns of pretraining and post-training biases suggest the use of a suboptimal readout and learning strategy, as biases were not always reduced through training. Indeed, for the easiest condition (500 ms), an expansion bias emerged with training, indicating that the VPL did not optimize performance. An optimal readout and learning strategy, such as readout reweighting, would be expected to improve perceptual sensitivity while decreasing biases to zero in all conditions, provided that observes received sufficient training (Petrov, Dosher, & Lu, 2005). In the following section, we use computational modeling to evaluate various readout and learning algorithms based on their ability to replicate our experimental findings.

### Computational model

The psychophysical results presented in the previous section reveal the following pattern of perceptual effects (Figure 2). 1) In all conditions, observers showed strong contraction bias before training. 2) Training improved motion discrimination on average. 3) Perceptual biases changed with training differently in the three conditions. In particular, in the most difficult condition (150-ms duration), the contraction bias remained after training. In the medium difficulty condition (350 ms), biases were typically decreased. In the easiest condition (500 ms), a large expansion bias emerged with training.

To gain insight into how training improved performance and altered the perceptual biases of the human observers, we developed a neural network model that, similar to area MST in the visual cortex, had an asymmetrical sensory representation of the two stimulus conditions (contracting vs. expanding optic flow). The two sensory populations ($x_{i}^{e}$ and $x_{i}^{c}$) projected to a single readout neuron that outputs the decision variable (here, expansion or contraction decision) (Figure 1A). The scalar output of the readout neuron was compared in the two stimulus conditions (i.e., expanding vs. contracting optic flow), and the type of optic flow in each trial was determined based on a decision criterion (*c_d_*). If the decision neuron fired larger than *c_d_*, the input stimulus would be determined as an expanding stimulus and contracting otherwise. Previous physiological studies reported that the majority of neurons in area MST are tuned to expansion optic flow. Specifically, Saito et al. (1986) reported a ratio of 70% expansion-tuned cells (Saito et al., 1986), Graziano et al. (1994) reported 84% (Graziano et al., 1994), and Heuer and Britten (2004) reported 87%. Given these varying ratios in the literature, we chose an 80% ratio in our computational model as an averaged representation of the previous reports, indicating that 80% of the sensory population in our model was set to be selective for expansion stimuli. The tuning properties of the artificial neurons were fixed and did not change during training. The only trainable components of the model were the readout weights, which set the connection strength between the sensory neurons and the readout neuron, as well as the decision criterion (*c_d_*). The learning rules only modulated the readout weight and/or the decision criterion throughout training. The equation below summarizes the model:

$$\begin{array}{c} y=\sigma \left(\sum_{i=1}^{M_{e}}w_{i}^{e}x_{i}^{e}+\sum_{i=1}^{M_{c}}w_{i}^{c}x_{i}^{c}+c_{d}\right). \end{array}$$

The goal was to determine which learning rule could best explain and reproduce observers’ behavior during the pretraining, training, and post-training phases. Before simulating the perceptual training experiment (our psychophysics experiments), we pretrained the neural network model to simulate the pre-experiment condition of the human observers. To achieve this goal, we trained the model exclusively with high-strength optic flow stimuli, under the assumption that the human visual system is generally adapted to high-coherence optic flow encountered in daily life. Thus, the preliminary training phase was conducted using very high coherence level stimuli ranging from 0.5 to 1.0.

We investigated two learning rules during the initial training phase:

(1) Optimal readout training: This learning rule adjusted the readout weights of individual sensory neurons (*w_i_*) as well as the decision criterion (*c_d_*) to achieve optimal performance.
(2) Decision criterion modulation: This learning rule only adjusted the decision criterion while keeping the sensory readout weights unchanged.

After the preliminary training, we evaluated the pretrained model using stimuli with varying coherence levels (0.15 to 0.65), to mimic the pretraining test phase of our experiment. To simulate the three different stimulus durations used in the psychophysics experiments, we scaled the coherence levels by three distinct factors (0.3, 0.6, and 1.0). This scaling was done to incorporate the influence of duration in the model, as suggested previously (Law & Gold, 2008).

**Figure 4.**
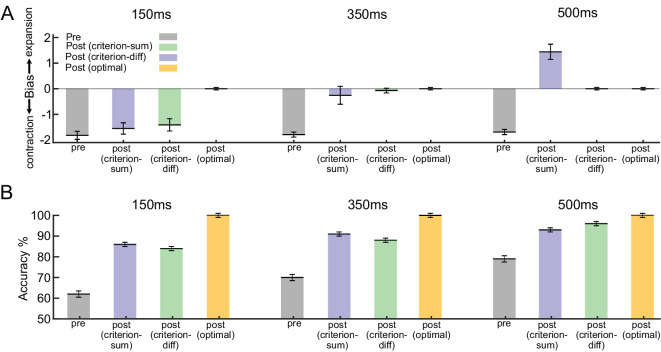
Comparison of the bias and discrimination accuracy of the model using different strategies during training. (**A**) During the preliminary training phase, the model was trained with decision criterion strategy modulation with high strength optic flow stimuli. The model showed significant bias toward contraction, similar to observer behavior (gray bars). After retraining with low-strength stimuli, the decision criterion model using the sum of population firing (criterion-sum, blue bars) could reproduce human experiment. However, both the decision criterion model that used the subtraction of responses from the two subpopulations reaching the downstream readout (criterion-diff, green bars) and the optimal model could not replicate the observers' behavior (optimal, yellow bars). (**B**) After the training, the discrimination accuracy (% correct) of all models was improved in all conditions. The accuracy of the optimal model always reached 100% after training. Error bars show the standard error of the mean (SEM).

When we used the optimal learning strategy during the preliminary training phase, the model showed no perceptual bias when tested with low strength stimuli. This finding was inconsistent with the human observers’ pretraining contraction bias. In contrast, once the model was trained with the decision criterion modulation strategy, it consistently showed a significant contraction bias across all levels of difficulty. This outcome closely resembled the results obtained in the human experiment (Figure 4A, gray bars). These findings suggest that, with an asymmetric sensory population, a simple and suboptimal readout approach (i.e., modifying only the decision criterion) can effectively account for the initial contraction bias observed in our psychophysics observations.

Next, we simulated the nine-session training phase of our psychophysics experiments by retraining the model using low coherence stimuli ranging from 0.2 to 0.3. We specifically chose this low coherence range to mimic the two-down–one-up staircase procedure used during the nine-session training phase of the human experiment. This procedure required the observers to concentrate on a limited coherence range centered around their perceptual threshold. Again, note that the model can be trained for this phase either by using the optimal learning strategy or decision criterion modulation (criterion-sum model: the decision criterion is determined by the sum of the population responses conveyed to the downstream readout; criterion-diff model: the decision criterion is set by subtracting responses from two subpopulations before they reach the downstream readout). Finally, similar to the post-training test in the human experiment, we tested the models again with the same range of coherences we used in the pretraining phase.

After the training, all models exhibited improved discrimination accuracy across all conditions (Figure 4B). However, the model using the optimal learning strategy displayed no bias in any of the conditions after training (Figure 4A). The decision criterion modulation (criterion sum), in contrast, could fully reproduce the human biases: For the difficult condition, there was a slight reduction of contraction bias but the bias remained significantly toward contraction. For the moderate condition, the model had no significant bias after training. For the easy condition, significant bias toward expansion emerged after training. Notably, this expansion bias only occurred when all neurons in the population had the same readout weight ($w_{i}^{e}=w_{i}^{c}=w$); that is, the downstream readout neuron received a sum of their firing. This key observation implies that the putative downstream readout neurons assign equal importance to each sensory neuron from which they receive information. An alternative approach, in which the two populations had equal but opposite-signed readout weights ($w_{i}^{e}=-w_{i}^{c}=w$; i.e., the downstream readout neuron receiving a difference of their firing), did not result in the same expansion bias.

**Figure 5.**
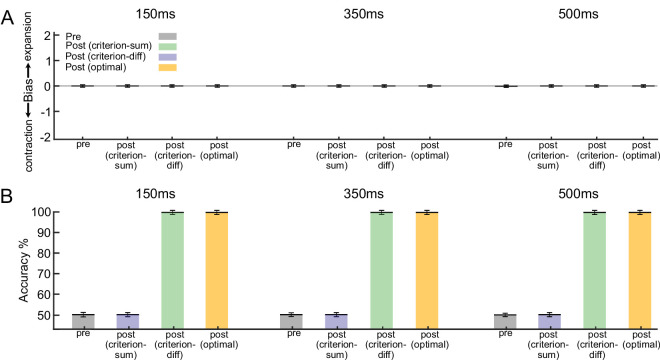
Comparison of the bias and discrimination accuracy of the model with a symmetrical population. (**A**) We trained the model with a symmetrical population using the decision criterion modulation strategy during the preliminary training phase. In the pretraining test, the model did not develop a bias in all conditions (gray bars). We then retrained the model with low strength stimuli using criterion-sum model (blue bars), criterion-diff model (green bars) or optimal readout model (yellow bars). The bias of all models remained at zero in all conditions in the post-training phase. (**B**) In the pretraining phase, the model's discrimination accuracy (% correct) remained at the chance level. After the training (post-training phase), there was no improvement in the performance of the criterion-sum model, while the discrimination accuracy of the criterion-diff and optimal readout models reached 100% in all conditions. Error bars show the standard error of the mean (SEM).

To determine whether the asymmetrical sensory representation was necessary to account for these results, we tested the model using a symmetric sensory population. The decision criterion modulation was applied during the preliminary training, followed by decision criterion modulation or optimal learning strategies in the training phase. In this case, the symmetric sensory population was unable to reproduce human behavior (Figure 5).

Humans can also enhance their performance by increasing the gain of sensory representations during training. This gain modulation strategy can be interpreted as an attention mechanism recruited during perceptual learning (Fox, Birman, & Gardner, 2023; Maunsell & Treue, 2006), but it can also involve other neuronal mechanisms that are not directly linked to attention.

**Figure 6.**
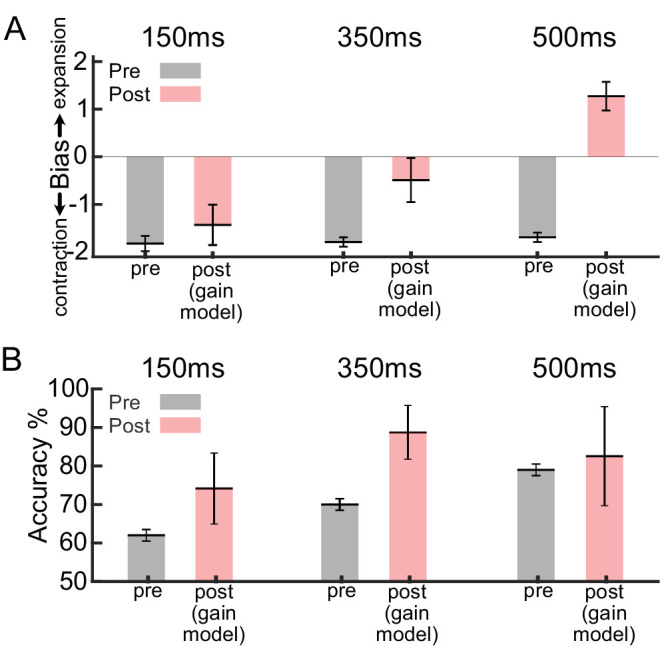
The bias and discrimination accuracy of the model using gain modulation strategy in the training phase. The perceptual training was modeled using the gain modulation strategy. After 50 epochs of training, the model could reproduce human behavior for (**A**) bias and (**B**) accuracy. Gray bars indicate the models’ results in pretraining, and red bars in post-training using gain modulation strategy. Error bars show the standard error of the mean (SEM).

Therefore, instead of altering the decision criterion, an alternative approach to training could involve adjusting the gain of sensory responses to align with the decision criterion value. We tested this strategy by introducing multiplicative gains to the sensory readout (*g^e^*and *g^c^*) (see Methods and Equation 3). Instead of optimizing the readout weights or the decision criterion, we assessed the learning dynamics of adjusting these sensory gains during training. As shown in Figure 6, the gain modulation strategy could reproduce our observed bias and accuracies across the three difficulty conditions. These findings indicate that modulating the decision criterion (which is also equivalent to an additive gain modulation of neurons' responses; see the Discussion for more details) and multiplicative gain modulation can reproduce our psychophysical results. It is important to emphasize that both the decision criterion and gain modulation are suboptimal learning strategies because they do not involve optimal tuning of all weight values of the model.

## Discussion

Practicing perceptual tasks has been shown to enhance performance, and these improvements have primarily been attributed to the adaptation of existing sensory representations or their readouts to the specific training task (Karni & Sagi, 1991; Lu & Dosher, 2022; Sotiropoulos et al., 2011; Wenliang & Seitz, 2018). These adaptations are commonly assumed to be optimal, in the sense that the sensory and/or sensorimotor synaptic connections are expected to undergo changes that optimize task accuracy. However, in our study, we sought to challenge this optimality assumption by investigating perceptual learning within a paradigm that relied on an asymmetric sensory representation. The over-representation of specific classes of stimuli in visual cortex enables the possibility of a suboptimal readout solution, which is not viable with a symmetric sensory representation (Figure 1). For these stimuli, we find that observers exhibit patterns of biased perception that change with training (Figure 2) in a manner that depends on task difficulty (Figure 3).

Through computational modeling, we show that a simple adjustment of the decision criterion adequately accounted for the initial and the training induced biases of the observers (Figure 4). Notably, an optimal learning rule failed to explain the observed biases (Figures 5, 6). Considering the prevalence of asymmetric sensory representations in the cortex, our findings make critical contributions to our understanding of learning within the visual system. By uncovering the role of decision criterion modulation in perceptual learning, particularly in the context of asymmetric representations, we challenge the prevailing assumptions regarding optimal adaptation of sensory and sensorimotor connections.

### Representational asymmetries in the visual cortex

The primate visual cortex consists of many different brain regions, most of which respond specifically to particular stimulus features. In most of these regions, the encoding of stimulus features is not uniform but rather biased toward specific stimulus properties. These biases can take the form of higher firing rates, narrower tuning curves, or non-uniform distributions of stimulus preferences. Asymmetries can also change across retinal locations (Albright, 1989; Ponce, Hartmann, & Livingstone, 2017; Sasaki et al., 2006).

Probably the best-known example is the cardinal bias found for orientation selectivity in the primary visual cortex (V1) (DeValois, 1982; Li, Peterson, & Freeman, 2003): There are more V1 neurons that prefer horizontal or vertical orientations than oblique ones. This asymmetry might coexist with a radial organization, in which the most common preferred orientation varies with receptive field position (Sasaki et al., 2006). In either case, the result is that the V1 population response is greater for some orientations than for others, setting up the conditions for learning to exploit changes in decision criteria. This would seem to be important for the field of VPL, given that so many studies use oriented stimuli (Song et al., 2010).

Other biases that have been detected neurophysiologically include biases for dark stimuli (Komban et al., 2014; Yeh, Xing, & Shapley, 2009), “daylight” colors (Lafer-Sousa, Liu, Lafer-Sousa, Wiest, & Conway, 2012), near disparities (DeAngelis & Uka, 2003; Tanabe, Doi, Umeda, & Fujita, 2005), small motion stimuli (Liu, Haefner, & Pack, 2016; Tsui & Pack, 2011), concave surfaces (Verhoef, Vogels, & Janssen, 2012), and curved shapes (Pasupathy & Connor, 2002). There are also entire brain regions dedicated to specific categories of stimuli, such as motion, color, faces, and food, and it is conceivable that observers rely on these asymmetries by changing their readout strategies during VPL (Bakhtiari, Awada, & Pack, 2020; Chen, Cai, Zhou, Thompson, & Fang, 2016; Liu & Pack, 2017).

These cortical asymmetries tend to reflect ecological or evolutionary experience (Brenner & Rauschecker, 1990; Mineault et al., 2012). The strong preference for expansion over contraction optic flow is likely linked to the visual experience of frontal-eyed animals during locomotion, and the bias for small moving stimuli has long been associated with the function of separating objects from the background (Lettvin, Maturana, McCulloch, & Pitts, 1959; Liu et al., 2016; Tadin et al., 2019). Cardinal orientations are apparently overrepresented in natural images (Girshick, Landy, & Simoncelli, 2011), and the bias toward curved or circular stimuli in the midlevel cortex could be a part of a network for face processing in the higher level cortex (Ponce, Hartmann, & Livingstone, 2017). Our findings suggest that the brain readily leverages these asymmetries during VPL, highlighting the importance of neurophysiological considerations in visual learning (Bakhtiari et al., 2020).

In this regard, it is worth considering the influence of natural stimuli outside the laboratory during learning experiments. For experiments that span several days, exposure to the natural world would be expected to counteract or reinforce VPL, depending on the nature of the stimuli and their representation in cortex.

It is also important to highlight that the ability to leverage preexisting representational biases might also be influenced by the nature of the task. Specifically, our study used a coarse optic flow discrimination task where the two optic flow conditions were distinctly different. Such a task condition could potentially influence the choice between optimal and suboptimal learning strategies. Future research should revisit this inquiry within the context of a fine discrimination task, where the two stimulus conditions are closer (e.g., expanding versus contracting spiral motion).

Although the cortical asymmetries of specific visual representations, as reported elsewhere in this article, have been studied extensively, the behavioral outcomes stemming from these asymmetries need further investigation. It is expected that an over-representation of a stimulus in the cortex would result in enhanced discrimination accuracy for that stimulus. Yet, the impact of such over-representations on the subjective appearance of stimuli remains ambiguous. For instance, when presented with noisy and uncertain stimuli, human observers perceive orientations that deviate from the actual horizontal and vertical orientations—orientations that are over-represented in the visual cortex (Szpiro, Burlingham, Simoncelli, & Carrasco, 2022). This observation aligns with the contraction bias noted in our study, which suggests a perceptual deviation away from the over-represented stimulus condition. Conversely, other over-represented stimuli, like faces, correlate with increased detection rates under noisy conditions, as seen in phenomena like face pareidolia (Wardle, Taubert, Teichmann, & Baker, 2020). The discrepancies in observed effects might stem from inconsistencies in the task paradigms across different studies. A comprehensive comparison of these over-represented stimuli and their behavioral manifestations within a consistent task framework is a pivotal direction for future.

### Comparison with previous psychophysical results

Some of the abovementioned cortical asymmetries have likely perceptual consequences. For orientation, there is the oblique effect (Appelle, 1972), in which human and animal observers are more sensitive to horizontal and vertical orientations than to others. As with cortical responses, human psychophysical observers are more sensitive to dark stimuli than to light stimuli (Komban et al., 2014). The cortical preference for small moving stimuli is associated with a wide range of perceptual phenomena presumably related to figure–ground segregation (Tadin, 2015). The over-representation of foveal stimuli in most of retinotopic cortex has been linked to perceptual effects that occur during eye movements (Richard, Churan, Guitton, & Pack, 2009). And the over-representation of entire classes of stimuli, such as faces, is thought to be partly responsible for illusory perceptual phenomena (i.e., pareidolias). Some of these biases can be decreased or abolished with training (Bakhtiari et al., 2020; Furmanski, Schluppeck, & Engel, 2004).

An example of this phenomenon was recently presented in work by Szpiro, Burlingham, Simoncelli, & Carrasco (2022), where it was demonstrated that perceptual learning of orientation discrimination amplified the preexisting bias toward cardinal orientations. They modeled the post-training improvement in perception by proposing an increased gain of neurons encoding the task stimuli, which could also reproduce their observed humans’ biases. However, an important distinction between their study and ours lies in the fact that their task stimuli consisted exclusively of under-represented stimuli, which could lead to different perceptual biases.

The consequences of these asymmetries are seldom considered in VPL studies, most of which are concerned with changes in sensitivity. Indeed, many models assume that decision criteria are set optimally, in an unbiased manner (Petrov et al., 2005). Nevertheless, in detection paradigms, people often alter their decision criteria, even though this strategy is not optimal (Wenger, Copeland, Bittner, & Thomas, 2008), and this factor can result in biased perceptual responses (Seitz, Nanez, Holloway, Koyama, & Watanabe, 2005). Our findings extended this strategy to a discrimination task that involved asymmetrical cortical representations of two distinct stimulus conditions. In this particular case, humans seem to be able to transform the discrimination task into a detection task, by aiming to detect the overrepresented stimulus condition (here, the expansion optic flow).

Some previous work has examined the effects of asymmetries in the design of perceptual learning tasks. In these experiments, asymmetries are created by presenting one stimulus more often than another, or by providing false or irrelevant feedback (Herzog, Ewald, Hermens, & Fahle, 2006; Herzog & Fahle, 1999; Seitz et al., 2005). In these cases, observers seem to respond by altering their decision criteria. Although changes in sensitivity and in criterion often occur simultaneously (Wenger et al., 2008), they have different characteristics, the most notable of which is the faster dynamics of changes in criteria (Aberg & Herzog, 2012).

Additionally, it is important to highlight some limitations in our study owing to the remote nature of our experiment's implementation. In this study, we conducted psychophysical experiments remotely, with participants using their personal computers at home, controlled by a browser-based program. Although this approach allowed for a larger and more diverse participant pool, it also introduced certain limitations that are worth consideration. First, the use of personal computers means that there is inherent variability in the hardware and software configurations across participants. This variability includes differences in display types, sizes, and refresh rates, which can impact the consistency of stimulus presentation and task performance. Although stimulus parameters were calibrated to each participant's screen size, this may not fully account for variations in display quality and resolution. Additionally, although the viewing distance was adjusted according to individual screen sizes, controlling factors like ambient lighting and seating posture, which can affect perceptual performance, was challenging in the home environment, potentially impacting environmental validity across participants. However, it is important to emphasize that these factors should not systematically affect our results because they were common to all participants across all three conditions (150-ms, 350-ms, and 500-ms stimulus durations). For this reason and to ensure consistency across conditions, we analyzed three key metrics that assess learning effects for each participant within each condition: 1) changes in psychometric threshold before and after training, 2) alterations in the slope of the psychometric curve before and after training, and 3) shifts in the lapse rate over the training period. Subsequently, we conducted an analysis of variance on these metrics to identify any condition-related disparities. The results indicated no significant differences among the conditions (*p* > 0.05, analysis of variance). Further studies could explore ways to standardize the at-home experimental conditions or consider alternative methods to address these limitations.

### Implications for learning in the cortex

In most psychophysics experiments, it is challenging to differentiate between different strategies and learning rules. A change in decision criterion would likely yield results similar to improved attentional focus, which is known to be crucial for learning (Szpiro & Carrasco, 2015). Specifically, a modulatory attentional effect that changes the excitability of sensory neurons’ responses could be similar to the decision criterion modulation observed in our study. Interestingly, previous research has reported biases in perceptual responses to attended stimuli versus unattended stimuli (Itthipuripat, Chang, Bong, & Serences, 2019), showing a perceptual effect similar to the observations in this study.

Despite the challenges of distinguishing between different learning strategies, our results are in conflict with the prevailing notion that performance is optimized through learning. This notion may hold true when using stimuli with symmetric representations and investigating learning solely based on perceptual sensitivity (Petrov et al., 2005). However, under asymmetric stimulus representations, which are common in the cortex, as mentioned earlier, suboptimal and easier solutions may be adopted (Liu, Dosher, & Lu, 2015). Our study shows that an optimal learning algorithm, such as gradient descent, does not automatically discover the suboptimal and easier solution under asymmetric representations.

Fitzgerald et al. (2013) raised an intriguing possibility that observers may tend to create low-dimensional representations of the task stimuli. This strategy enables observers to introduce their own representational asymmetries, even where the underlying sensory representations of the task stimuli are symmetric. The creation of such low-dimensional and asymmetric representations, as demonstrated in this study, facilitates a simpler learning process for the stimulus–response mapping. Therefore, learning strategies that exploit asymmetric representations may potentially remain latent in numerous VPL experiments, including those that do not rely explicitly on the intrinsic representational asymmetries of the visual cortex.

For certain kinds of learning tasks, asymmetric representation could be useful. For applications of the VPL in particular, changes in sensitivity often require prolonged training and fail to generalize beyond the trained stimulus, limiting their utility for therapeutic purposes or other applications. Our results, along with previous findings (Aberg & Herzog, 2012) suggest that decision criteria can be adjusted more rapidly, often within a single session, although the learning effects might also be more transient (Aberg & Herzog, 2012). One goal for future research could therefore be to exploit representational asymmetries in the brain to develop faster learning (Pandey, Neupane, Vaidya, Adhikary, & Pack, 2022).

### Comparison with previous computational models

Previously proposed theories of VPL have primarily suggested optimal changes either in the sensory representations (referred to as retuning) (Karni & Sagi, 1991; Wenliang & Seitz, 2018) or in the readout of sensory neurons (referred to as reweighting) (Lu & Dosher, 2022; Sotiropoulos et al., 2011). However, these theories have assumed homogeneous sensory representation of task stimuli, whereas, as we showed elsewhere in this paper, an asymmetric representation enables a simpler learning strategy that does not align with the existing literature. Recent studies have provided computational evidence demonstrating that artificial neural networks also display similar representational asymmetries, as they mirror the biases present in their training data (Benjamin, Zhang, Qiu, Stocker, & Kording, 2022). Learning algorithms capable of leveraging these representational asymmetries may bring artificial neural networks closer to capturing human learning dynamics.

For a naïve observer, before training, the readout weights of sensory neurons are not inherently different owing to the unfamiliarity with the task and the absence of a priori assumptions regarding the relative importance of these neurons in the task (Wenliang & Seitz, 2018). In the case of asymmetric representation of task stimuli, as discussed earlier, the initial equal readout weights are effective enough to solve the task (Figure 1). The key question is whether humans would optimize learning by adjusting the readout weights of all sensory neurons, deviating from equal readout weights, or if they would maintain the suboptimal strategy of equal readout weights and enhance performance solely by modulating the decision criterion. Our findings suggest that, when feasible, humans learn to improve task performance by solely adjusting the decision criterion, without modifying the readout weights. It is also justifiable from a computational complexity perspective as it is more efficient to find and store the optimal value of one parameter (i.e., the decision criterion) rather than optimizing a large number of sensory readout weights. This becomes more critical for multitask learning. Previous work on few-shot learning in artificial neural networks (Triantafillou, Larochelle, Zemel, & Dumoulin, 2021) has suggested the advantages of a similar approach in machine learning, namely, keeping the feedforward connection weights stable and adjusting the bias and scale of the readout from each layer for every new task. Although our focus in this study was primarily on sensorimotor readout, this approach could potentially be applicable to multiple layers.

Furthermore, our observed modulation of the decision criterion can be interpreted as a global change in the firing rate of sensory neurons. Specifically, a decrease (or increase) in the decision criterion in our model corresponds to a global increase (or decrease) in the firing rate of sensory neurons (see Equation 3). We also showed that learning via adjusting a multiplicative gain could reproduce the biases observed in humans. These results suggest that the proposed role of decision criterion or gain modulation in perceptual learning could be implemented through an additive or multiplicative top-down modulation of the global firing rate of sensory neurons. Interestingly, a previous neural network model of perceptual learning (Herzog & Fahle, 1998) highlighted the significance of top-down modulation signals in capturing the dynamics of perceptual learning. This observation implies that, unlike gradient descent, more biologically plausible learning algorithms that rely on top-down modulation of neurons’ activation, such as target propagation (Lee, Zhang, Fischer, & Bengio, 2015) and its more recent variants (Meulemans, Zucchet, Kobayashi, von Oswald, & Sacramento, 2022), may be able to reproduce our observations. Despite converging to similar solutions in the steady state (Meulemans et al., 2022), the trajectory of reaching the final solution may differ among these learning algorithms that make comparisons with the dynamics of human learning worthwhile.

In our modeling experiments, we trained all variants of the model for a fixed number of epochs, considering the time constraints in human psychophysics experiments. After this fixed training period, we compared the performance of different learning strategies. However, we also noticed that longer training times for two of our learning strategies, namely, decision criterion modulation and gain modulation, resulted in distinct bias patterns. Unlike decision criterion modulation, extending the training period with gain modulation led to an expansion bias across all task conditions (easy, medium, and difficult). Currently, our psychophysics data are insufficient to differentiate between these two possibilities, because longer training periods are necessary for the more challenging conditions.

## Conclusions

We demonstrated that, when relying on asymmetric sensory representations, humans use a simple readout and learning strategy to improve their perceptual performance. This strategy cannot be explained by the optimal learning algorithms proposed in previous studies. Considering the widespread representational asymmetries in the cortex, our findings carry significant implications for learning mechanisms in cortical processing. Future research should explore a broader range of learning algorithms, particularly those that incorporate top-down feedback modulations, to examine their capacity to replicate the learning dynamics and biases observed in humans.
